# Supplementary material for: Assessment of the Genetic Diversity of Rice Germplasms Characterized by Black-Purple and Red Pericarp Color Using Simple Sequence Repeat Markers
Source: Plants (Basel). 2019 Nov 4;8(11):471. doi: 10.3390/plants8110471 (PMC6918417; doi:10.3390/plants8110471)
Supplement: Supplementary file 1 [file plants-08-00471-s001.pdf]

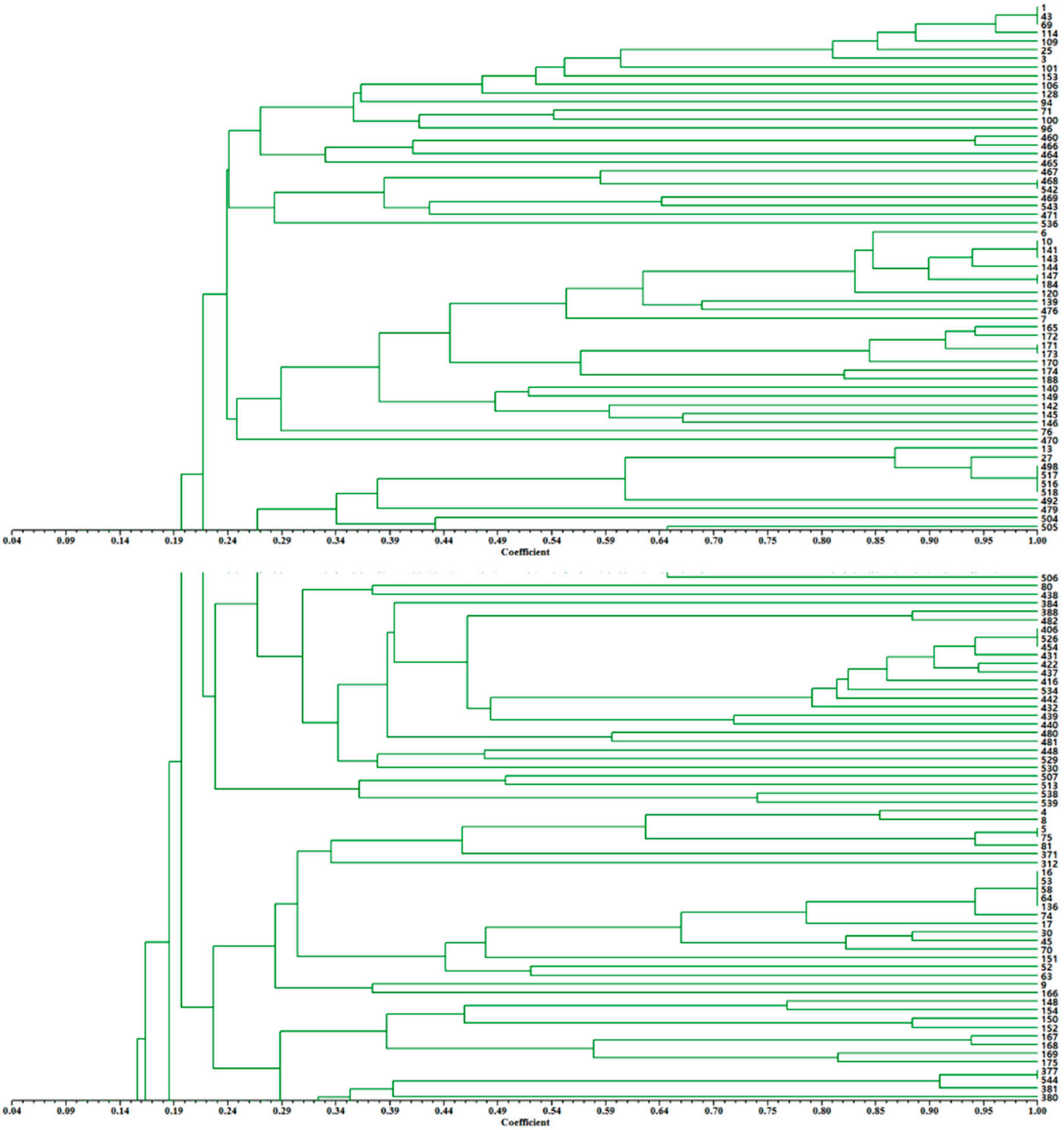

Supplement Figure 1. Dendrogram of 548 rice varieties based on 16 SSR markers. The scale at the bottom is Jaccard's coefficient of similarity(continue).

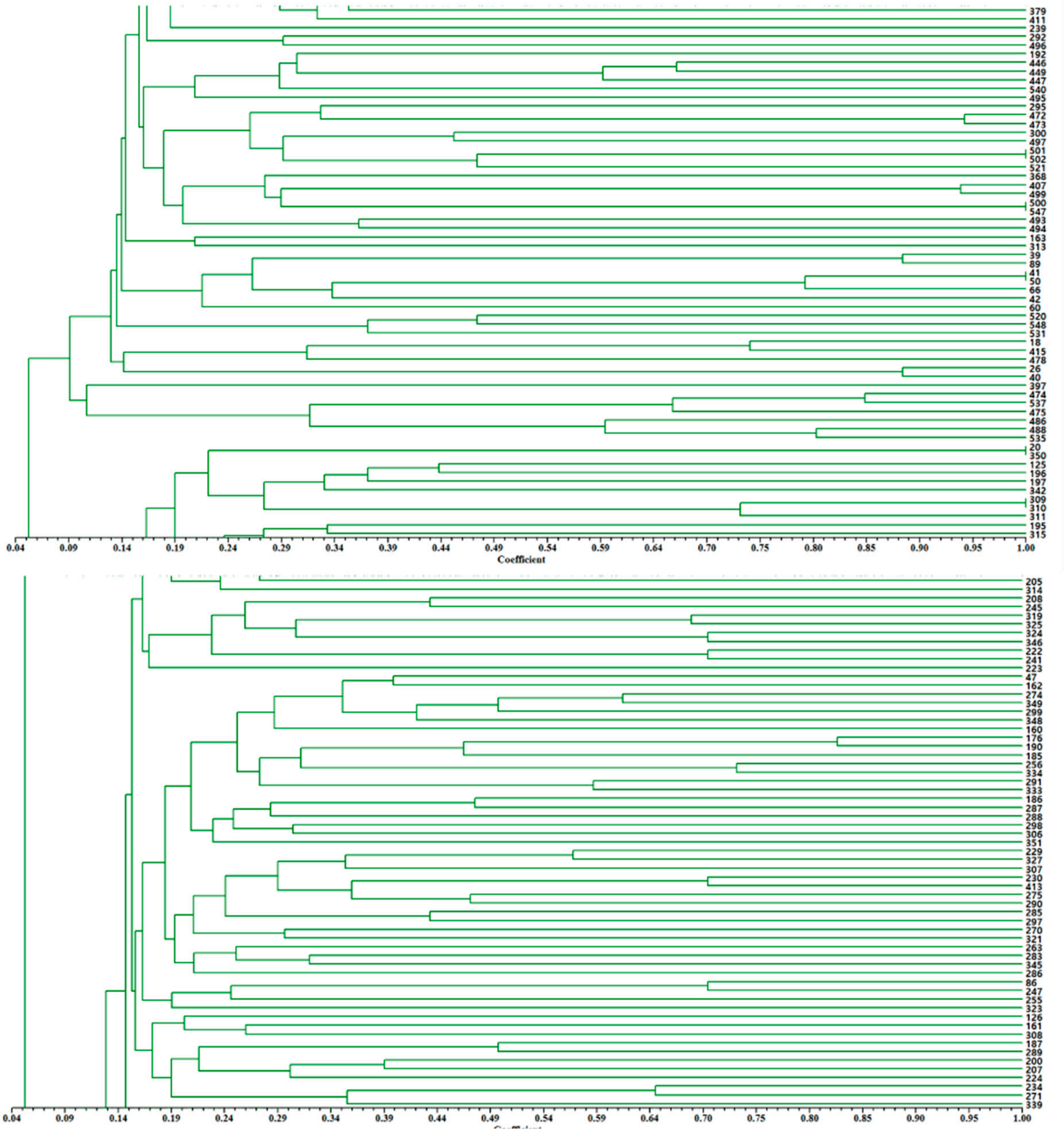

Supplement Figure 1. Dendrogram of 548 rice varieties based on 16 SSR markers. The scale at the bottom is Jaccard's coefficient of similarity(continue).

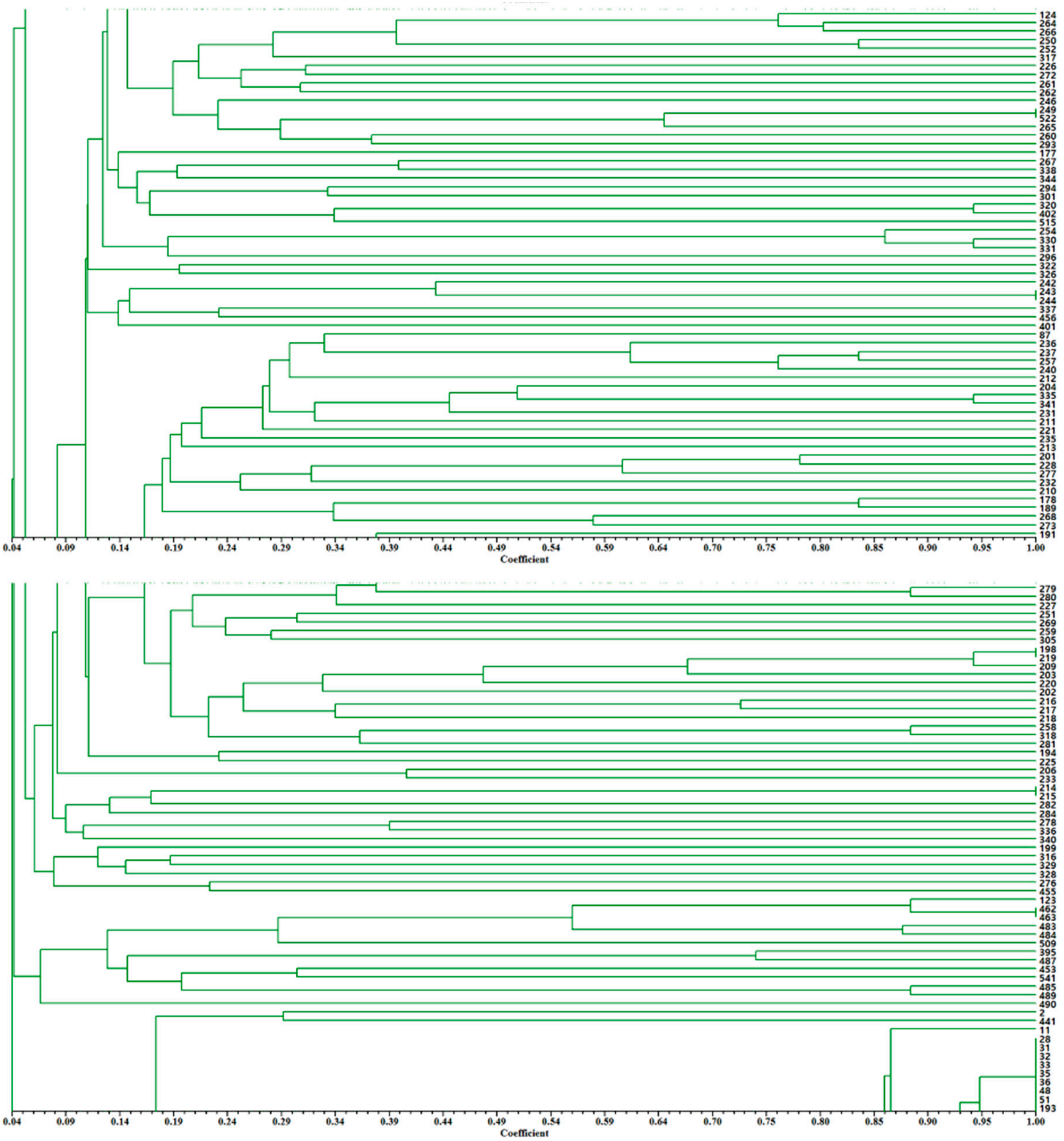

Supplement Figure 1. Dendrogram of 548 rice varieties based on 16 SSR markers. The scale at the bottom is Jaccard's coefficient of similarity(continue).

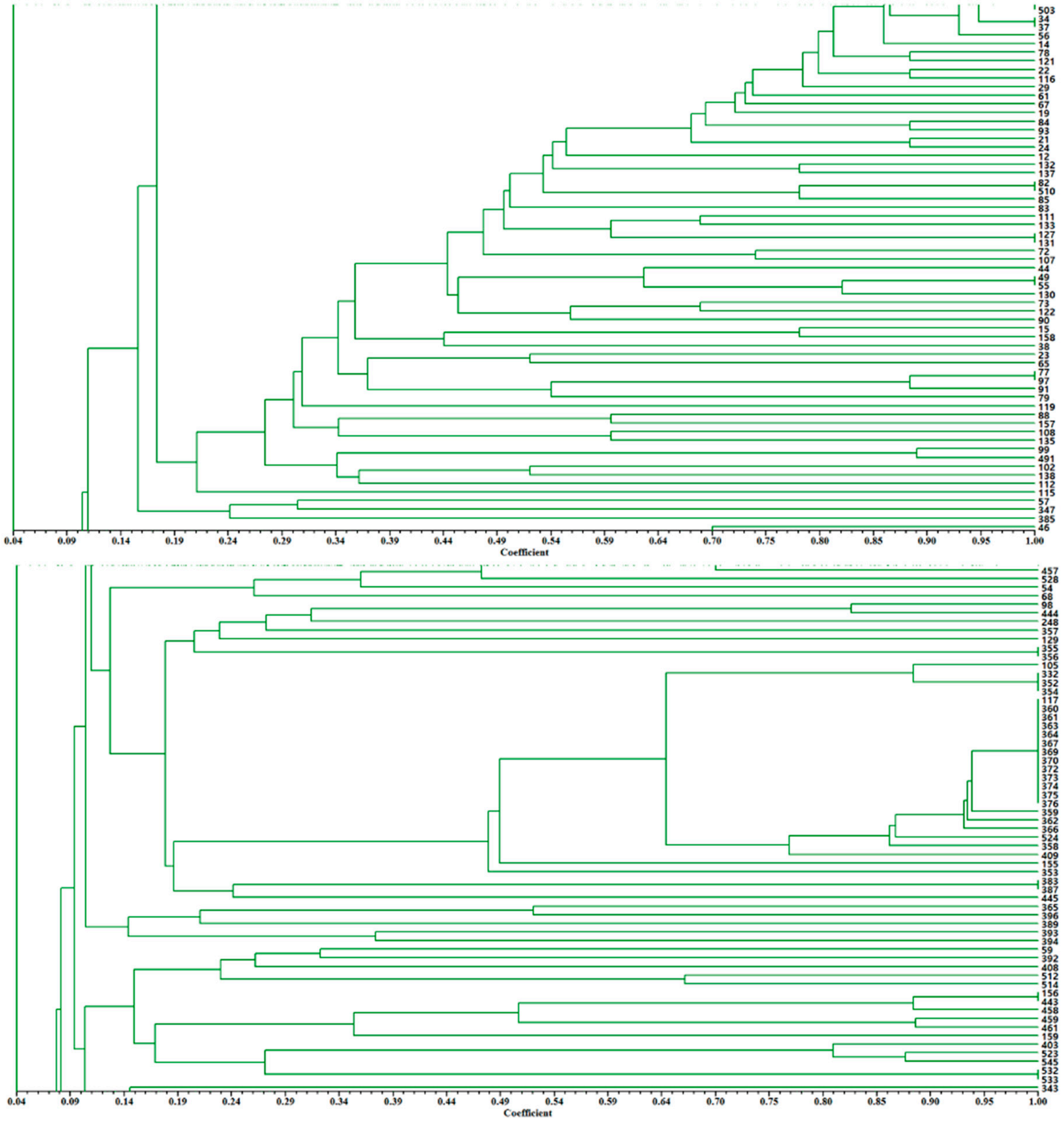

Supplement Figure 1. Dendrogram of 548 rice varieties based on 16 SSR markers. The scale at the bottom is Jaccard's coefficient of similarity(continue).

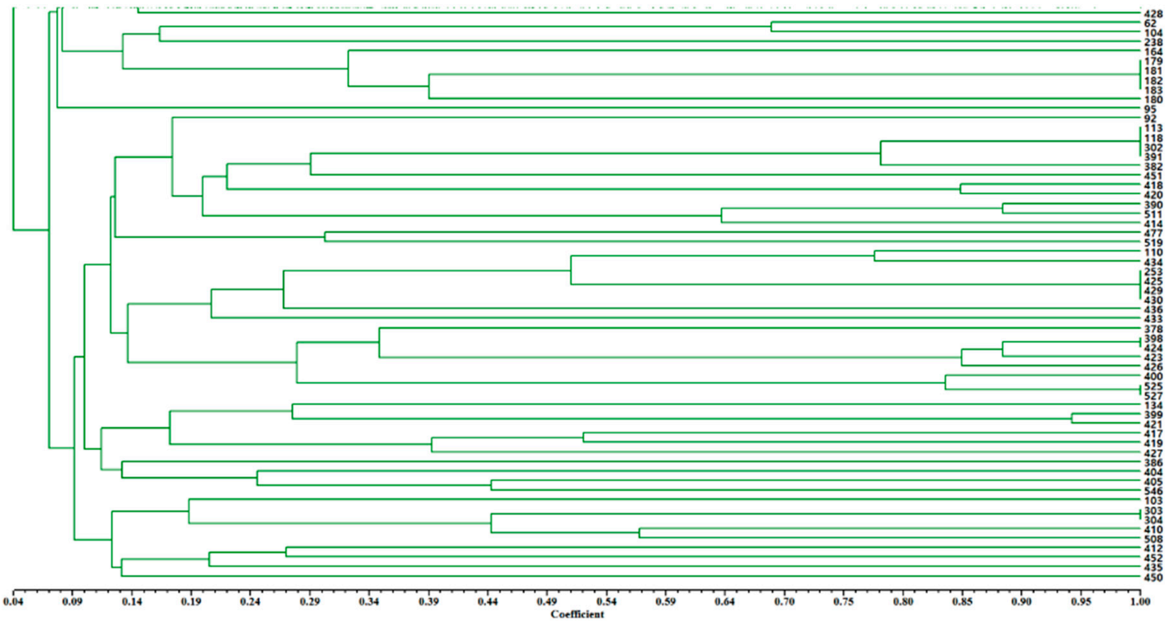

Supplement Figure 1. Dendrogram of 548 rice varieties based on 16 SSR markers. The scale at the bottom is Jaccard's coefficient of similarity(continue).

Supplementary Table 1. List of rice cultivars and germplasms assayed for genetic characterization using microsatellite markers.

No.	Source of seed	Country code	Country	Cluster
001	Jilin collection #7(Black Aromatic Waxy 1)	CHN	China	Ⅰ-1
002	Jilin collection #6(Black Aromatic3)	KOR	Korea	Ⅲ
003	Jilin collection #5(Black Aromatic 2)	CHN	China	Ⅰ-1
004	Jilin collection #4(Black Aromatic 1)	CHN	China	Ⅰ-1
005	Jilin collection #8(Black Aromatic Waxy 2)	CHN	China	Ⅰ-1
006	Heimi F-8	CHN	China	Ⅰ-1
007	Mixed in Guongzuonmi #12	CHN	China	Ⅰ-1
008	Jilin collection #3(Heimi 3)	CHN	China	Ⅰ-1
009	Jilin collection #2(Heimi 2)	CHN	China	Ⅰ-1
010	Nanjing Xiang dao	CHN	China	Ⅰ
011	Mixed in Guongzuonmi #13	CHN	China	Ⅲ
012	ChoongBuk Collection	KOR	Korea	Ⅲ
013	Mixed in Guongzuonmi #8	CHN	China	Ⅰ-1
014	JBR # 12	IDN	Indonesia	Ⅲ
015	Beijing collection #47	CHN	China	Ⅲ
016	Beijing collection #9	CHN	China	Ⅰ-1
017	Guongzuonmi(Miyazaki Univ.)	CHN	China	Ⅰ-1
018	Hei Mi	CHN	China	Ⅰ-1
019	Beijing collection #8	CHN	China	Ⅲ
020	Mixed in Jindo Collection #18	KOR	China	Ⅰ-3
021	Beijing collection #15	CHN	China	Ⅲ
022	Beijing collection #16	CHN	China	Ⅲ
023	Beijing collection #18	CHN	China	Ⅲ
024	Beijing collection #6	CHN	China	Ⅲ

025	Jindo Collection	KOR	China	Ⅰ-1
026	Jahyangdo	CHN	China	Ⅰ-1
027	Cambodia Coll.(Ta Khmau Market)	KHM	Cambodia	Ⅰ-1
028	Hei Bao	CHN	China	Ⅲ
029	Beijing collection #38	CHN	China	Ⅲ
030	Guongzuonmi Separated 1	CHN	China	Ⅰ-1
031	Jilin Heimi	CHN	China	Ⅲ
032	Beijing collection #14	CHN	China	Ⅲ
033	Abhyeulna	CHN	China	Ⅲ
034	Goheung Collection	KOR	Korea	Ⅲ
035	Waitou Murasaki Daikoku	JPN	Japan	Ⅲ
036	Beijing collection #13	CHN	China	Ⅲ
037	Cambodia Coll.(Takov Village)	KHM	Cambodia	Ⅲ
038	Mixed in Jindo Collection #23	KOR	Korea	Ⅲ
039	Shanghai Collection #15	CHN	China	Ⅰ-1
040	Jahyangna	CHN	China	Ⅰ-1
041	Beijing collection #48	CHN	China	Ⅰ-1
042	Beijing collection #12	CHN	China	Ⅰ-1
043	Zaliang	CHN	China	Ⅰ-1
044	Mixed in Jindo Collection #1	KOR	Korea	Ⅲ
045	Beijing collection #3	CHN	China	Ⅰ-1
046	JBR # 7	IDN	Indonesia	Ⅲ
047	Shanghai Collection #12	CHN	China	Ⅰ-3
048	Beijing collection #7	CHN	China	Ⅲ
049	Beijing collection #17	CHN	China	Ⅲ
050	Mixed in Jindo Collection #11	KOR	Korea	Ⅰ-1
051	JBR # 4	IDN	Indonesia	Ⅲ

052	Hoijinheukmi	KOR	Korea	Ⅰ-1
053	Mixed in Jindo Collection #19	KOR	Korea	Ⅰ-1
054	Shanghai Collection #16	CHN	China	Ⅲ
055	Mixed in Jindo Collection #17	KOR	Korea	Ⅲ
056	JBR # 3	IDN	Indonesia	Ⅲ
057	Yeon Nong Heug #1	KOR	Korea	Ⅲ
058	Guongzuonmi(Kyushu Univ.)	CHN	China	Ⅰ-1
059	Beijing collection #42	CHN	China	Ⅲ
060	Beijing collection #37	CHN	China	Ⅰ-1
061	Beijing collection #44	CHN	China	Ⅲ
062	Heuk woo jom #1	KOR	Korea	Ⅲ
063	Shanghai Collection #19	CHN	China	Ⅰ-1
064	Hei Zuon Mi	CHN	China	Ⅰ-1
065	Beijing collection #5	CHN	China	Ⅲ
066	Beijing collection #21	CHN	China	Ⅰ-1
067	Beijing collection #22	CHN	China	Ⅲ
068	JUKU	JPN	Japan	Ⅲ
069	Shanghai Collection #17	CHN	China	Ⅰ-1
070	Shanghai Collection #6	CHN	China	Ⅰ-1
071	Mixed in Jindo Collection #21	KOR	Korea	Ⅰ-1
072	Beijing collection #26	CHN	China	Ⅲ
073	Beijing collection #39	CHN	China	Ⅲ
074	Hong Xue Nuo	CHN	China	Ⅰ-1
075	Beijing collection #1	CHN	China	Ⅰ-1
076	Hong Xue Nuo	CHN	China	Ⅰ-1
077	Mixed in Jindo Collection #15	KOR	Korea	Ⅲ
078	Beijing collection #11	CHN	China	Ⅲ

079	Beijing collection #29	CHN	China	Ⅲ
080	Mixed in Jindo Collection #2	KOR	Korea	Ⅰ-1
081	Mixed in Jindo Collection #16	KOR	Korea	Ⅰ-1
082	Beijing collection #43	CHN	China	Ⅲ
083	JBR # 9	IDN	Indonesia	Ⅲ
084	Beijing collection #31	CHN	China	Ⅲ
085	Beijing collection #41	CHN	China	Ⅲ
086	Beijing collection #20	CHN	China	Ⅰ-3
087	Lutao Heimi	CHN	China	Ⅰ-3
088	Mixed in Jindo Collection #7	KOR	Korea	Ⅲ
089	Cambodia Coll.(Wet Phnom Village)	KHM	Cambodia	Ⅰ-1
090	Beijing collection #2	CHN	China	Ⅲ
091	Mixed in Jindo Collection #12	KOR	Korea	Ⅲ
092	Beijing collection #19	CHN	China	Ⅳ-1
093	Beijing collection #30	CHN	China	Ⅲ
094	Mixed in Jindo Collection #22	KOR	Korea	Ⅰ-1
095	Jajin	CHN	China	Ⅲ
096	Mixed in Jindo Collection #20	KOR	Korea	Ⅰ-1
097	Mixed in Jindo Collection #13	KOR	Korea	Ⅲ
098	Touhuku 149	JPN	Japan	Ⅲ
099	Mixed in Jindo Collection #10	KOR	Korea	Ⅲ
100	Shanghai Collection #7	CHN	China	Ⅰ-1
101	Shanghai Collection #18	CHN	China	Ⅰ-1
102	Beijing collection #27	CHN	China	Ⅲ
103	Beijing collection #4	CHN	China	Ⅳ-2
104	Shanghai Collection #10	CHN	China	Ⅲ
105	Unknown variety from China #1	CHN	China	Ⅲ

106	Shanghai Collection #3	CHN	China	Ⅰ-1
107	Beijing collection #35	CHN	China	Ⅲ
108	Beijing collection #32	CHN	China	Ⅲ
109	Shanghai Collection #13	CHN	China	Ⅰ-1
110	Shanghai Collection #8	CHN	China	Ⅳ-1
111	JBR # 1	IDN	Indonesia	Ⅲ
112	Beijing collection #40	CHN	China	Ⅲ
113	JBR # 2	IDN	Indonesia	Ⅳ-1
114	Shanghai Collection #14	CHN	China	Ⅰ-1
115	JBR # 8	IDN	Indonesia	Ⅲ
116	Beijing collection #25	CHN	China	Ⅲ
117	Murasaki Ine	JPN	Japan	Ⅲ
118	Mixed in Jindo Collection #14	KOR	Korea	Ⅳ-1
119	Mixed in Jindo Collection #9	KOR	Korea	Ⅲ
120	Shanghai Collection #1	CHN	China	Ⅰ-1
121	Beijing collection #10	CHN	China	Ⅲ
122	Beijing collection #28	CHN	China	Ⅲ
123	JBR # 6	IDN	Indonesia	Ⅱ
124	Shanghai Collection #5	CHN	China	Ⅰ-3
125	Beijing collection #34	CHN	China	Ⅰ-3
126	Meragome	CHN	China	Ⅰ-3
127	Beijing collection #23	CHN	China	Ⅲ
128	Shanghai Collection #9	CHN	China	Ⅰ-1
129	Mixed in Jindo Collection #6	KOR	China	Ⅲ
130	Beijing collection #24	CHN	China	Ⅲ
131	JBR # 11	IDN	Indonesia	Ⅲ
132	Shanghai Collection #2	CHN	China	Ⅲ

133	Mixed in Jindo Collection #5	KOR	Korea	Ⅲ
134	Beijing collection #36	CHN	China	Ⅳ-1
135	Mixed in Jindo Collection #8	KOR	Korea	Ⅲ
136	JBR # 5	CHN	China	Ⅰ-1
137	Ryung Jin #1	CHN	China	Ⅲ
138	Beijing collection #49	CHN	China	Ⅲ
139	Mixed in Guongzuonmi #2	CHN	China	Ⅰ-1
140	Mixed in Guongzuonmi #3	CHN	China	Ⅰ-1
141	Mixed in Guongzuonmi #5	CHN	China	Ⅰ-1
142	Mixed in Guongzuonmi #10	CHN	China	Ⅰ-1
143	Guongzuonmi Separated 2	CHN	China	Ⅰ-1
144	Guongzuonmi Separated 3	CHN	China	Ⅰ-1
145	Kagosima Collection	CHN	China	Ⅰ-1
146	Mixed in Guongzuonmi #4	CHN	China	Ⅰ-1
147	Mixed in Guongzuonmi #9	CHN	China	Ⅰ-1
148	Mixed in Guongzuonmi #7	CHN	China	Ⅰ-1
149	Mixed in Guongzuonmi #1	CHN	China	Ⅰ-1
150	Mixed in Jindo Collection #4	KOR	China	Ⅰ-1
151	Mixed in Jindo Collection #3	KOR	China	Ⅰ-1
152	Meisanheiku	CHN	China	Ⅰ-1
153	Shanghai Collection #11	CHN	China	Ⅰ-1
154	Mixed in Jindo Collection #24	KOR	Korea	Ⅰ-1
155	Jilin collection #2(Heimi 1)	CHN	China	Ⅲ
156	Jilin collection #10(Heimi 1)	CHN	China	Ⅲ
157	Jilin collection #11(Heimi 1)	CHN	China	Ⅲ
158	Jilin collection #12(Heimi 1)	CHN	China	Ⅲ
159	Jilin collection #13(Heimi 1)	CHN	China	Ⅲ

160	MALAGKIT PIRURUTONG	PHL	Philippines	Ⅰ-3
161	NANTON 84	TWN	Taiwan	Ⅰ-3
162	LUA CHUA CHAN	TWN	Taiwan	Ⅰ-3
163	KHAO KAM	TWN	Taiwan	Ⅰ-1
164	KAM DO	IDN	Indonesia	Ⅲ
165	HILL PADI	MYS	Malaysia	Ⅰ-1
166	PADI SELASAH	IDN	Indonesia	Ⅰ-1
167	MITAK	IDN	Indonesia	Ⅰ-1
168	CHAKHAO	IDN	Indonesia	Ⅰ-1
169	PADI ITAM	MYS	Malaysia	Ⅰ-1
170	PADI ADONG DUMARAT	MYS	Malaysia	Ⅰ-1
171	PADI ARONG 1	MYS	Malaysia	Ⅰ-1
172	KETAN IRENG	IDN	Indonesia	Ⅰ-1
173	KU59	THA	Tailand	Ⅰ-1
174	KU72-3	THA	Tailand	Ⅰ-1
175	GIANTI	IDN	Indonesia	Ⅰ-1
176	K. BULU SOPONJONO	IDN	Indonesia	Ⅰ-3
177	KETAN BLAWU	IDN	Indonesia	Ⅰ-3
178	KETAN DJINTEN	IDN	Indonesia	Ⅰ-3
179	KETAN HIDEUNG	IDN	Indonesia	Ⅲ
180	NEP THAN	VNM	Vietnam	Ⅲ
181	NEP CAM	VNM	Vietnam	Ⅲ
182	PIRURUTONG	PHL	Philippines	Ⅲ
183	JANAWNA	THA	Tailand	Ⅲ
184	KHAO' NIAW DAM	THA	Tailand	Ⅰ-1
185	NIAW RAI 1	THA	Tailand	Ⅰ-3
186	AEN METAN	IDN	Indonesia	Ⅰ-3

187	DALAM HITAM	IDN	Indonesia	Ⅰ-3
188	KEAREN HITAM	IDN	Indonesia	Ⅰ-1
189	KETAN ARAM	IDN	Indonesia	Ⅰ-3
190	KETAN DJINTEN	IDN	Indonesia	Ⅰ-3
191	KETAN HITAM	IDN	Indonesia	Ⅰ-3
192	KETAN HITAM	IDN	Indonesia	Ⅰ-1
193	KETAN IRENG	IDN	Indonesia	Ⅲ
194	KETAN ITAM	IDN	Indonesia	Ⅰ-3
195	KETAN ITEM BIASA	IDN	Indonesia	Ⅰ-3
196	KETAN KUWULE	IDN	Indonesia	Ⅰ-3
197	KETAN MARI KANGEN	IDN	Indonesia	Ⅰ-3
198	KETAN MURENG	IDN	Indonesia	Ⅰ-3
199	LEKE MEE	IDN	Indonesia	Ⅰ-3
200	PADI HITAM	MYS	Malaysia	Ⅰ-3
201	PADI IRENG	MYS	Malaysia	Ⅰ-3
202	PARE HIDEUNG	IDN	Indonesia	Ⅰ-3
203	RUSIP	IDN	Indonesia	Ⅰ-3
204	LAWANG(GLUT.)	IDN	Indonesia	Ⅰ-3
205	MAITAMITAM(GLUT)	PHL	Philippines	Ⅰ-3
206	LELENG KADARO	PHL	Philippines	Ⅰ-3
207	PADI SIARANG	MYS	Malaysia	Ⅰ-3
208	PULU BALONG	IDN	Indonesia	Ⅰ-3
209	PULU BALONG	IDN	Indonesia	Ⅰ-3
210	PULUT HITAM	IDN	Indonesia	Ⅰ-3
211	PULUT HITAM	IDN	Indonesia	Ⅰ-3
212	SIPULUT BABADJA	IDN	Indonesia	Ⅰ-3
213	SIPULUT HITAM	IDN	Indonesia	Ⅰ-3

214	SIPULUT HITAM PENDEK	IDN	Indonesia	Ⅰ-3
215	PADI HITAM	MYS	Malaysia	Ⅰ-3
216	ARC 11700	IDN	Indonesia	Ⅰ-3
217	DAMNOEUB KHMAO	KHM	Cambodia	Ⅰ-3
218	KETAN HITAM	IDN	Indonesia	Ⅰ-3
219	KETAN ITEM-TEGAL ARUM	IDN	Indonesia	Ⅰ-3
220	LEUKAT ADANG	IDN	Indonesia	Ⅰ-3
221	PULUT HITAM	IDN	Indonesia	Ⅰ-3
222	PULUT HITAM	IDN	Indonesia	Ⅰ-3
223	JANGGUT BUGIS HITAM	IDN	Indonesia	Ⅰ-3
224	PADI HITAM MELAYU	MYS	Malaysia	Ⅰ-3
225	SIPULUT HITAM	IDN	Indonesia	Ⅰ-3
226	ANE BONAK	IDN	Indonesia	Ⅰ-3
227	KETAN HITAM-JALA BUTUN	IDN	Indonesia	Ⅰ-3
228	KLAN HAM	IDN	Indonesia	Ⅰ-3
229	LEKATAN HITAM	IDN	Indonesia	Ⅰ-3
230	MERAH GUJU	IDN	Indonesia	Ⅰ-3
231	SIHITAM	IDN	Indonesia	Ⅰ-3
232	ASE PULU BOLONG	IDN	Indonesia	Ⅰ-3
233	BIU 1	IDN	Indonesia	Ⅰ-3
234	PULUT BOLONG	IDN	Indonesia	Ⅰ-3
235	WULUMATA	IDN	Indonesia	Ⅰ-3
236	KETAN HITAM	IDN	Indonesia	Ⅰ-3
237	TININTA	PHL	Philippines	Ⅰ-3
238	PAEBIU NGGORUMI-RUMI	IDN	Indonesia	Ⅲ
239	PAEBIU SITORO	IDN	Indonesia	Ⅰ-1
240	PAEDAI NDOWATU 2	IDN	Indonesia	Ⅰ-3

241	PARE DOLO	IDN	Indonesia	Ⅰ-3
242	PULU LOTONG	IDN	Indonesia	Ⅰ-3
243	PULU LOTONG	IDN	Indonesia	Ⅰ-3
244	PULUT JIN	IDN	Indonesia	Ⅰ-3
245	PULUT LELENG	IDN	Indonesia	Ⅰ-3
246	PULUT LELENG MALLOYO	IDN	Indonesia	Ⅰ-3
247	SANTUBI	IDN	Indonesia	Ⅰ-3
248	TINGGA LOKO	IDN	Indonesia	Ⅲ
249	PULU BOLONG	IDN	Indonesia	Ⅰ-3
250	BLE NG TCHANG TE	THA	Tailand	Ⅰ-3
251	JA NO NAQ	THA	Tailand	Ⅰ-3
252	KAO SIYENG	THA	Tailand	Ⅰ-3
253	NIAW GAN-YAH	THA	Tailand	Ⅳ-1
254	BALI	IDN	Indonesia	Ⅰ-3
255	CHELOM	PAK	Indonesia	Ⅰ-3
256	CHELUM	PAK	Pakistan	Ⅰ-3
257	UNNAMED	CHN	China	Ⅰ-3
258	HITAM-BIRA MIT	IDN	Indonesia	Ⅰ-3
259	KOTU	IDN	Indonesia	Ⅰ-3
260	PULUT JIGA	IDN	Indonesia	Ⅰ-3
261	BALAKYAR	THA	Tailand	Ⅰ-3
262	NGACHEIK	THA	Tailand	Ⅰ-3
263	NGACHEIK	THA	Tailand	Ⅰ-3
264	NGACHEIK	THA	Tailand	Ⅰ-3
265	TAUNG PYONE	MMR	Myanmar	Ⅰ-3
266	TAUNGYO NGACHEIK	MMR	Myanmar	Ⅰ-3
267	TONKIN	VNM	Vietnam	Ⅰ-3

268	YWETPONE	IDN	Indonesia	I -3
269	HITAM BUBAT	IDN	Indonesia	I -3
270	KACIAK TARAM	IDN	Indonesia	I -3
271	SIPULUIK ARANG	IDN	Indonesia	I -3
272	BULU HITAM	IDN	Indonesia	I -3
273	KETAN IREN BERDESAN	IDN	Indonesia	I -3
274	ARC 15014	IDN	Indonesia	I -3
275	ARC 15172	IDN	Indonesia	I -3
276	ARC 15222	IDN	Indonesia	I -3
277	BALLATINAO(DIKET)	PHL	Philippines	I -3
278	TININTA(MALAGKIT)	PHL	Philippines	I -3
279	TAPOL(RED)	PHL	Philippines	I -3
280	KHAO TSIANG	THA	Tailand	I -3
281	GAM LIANG	THA	Tailand	I -3
282	GLAM	THA	Tailand	I -3
283	KHAO GAM	THA	Tailand	I -3
284	AEB368 RIP P TYPE ADT	MMR	Myanmar	I -3
285	CHAKHAO	MMR	Myanmar	I -3
286	CHAKHAOHUIKAP	MMR	Myanmar	I -3
287	MARAGADAN	PHL	Philippines	I -3
288	MARAGADAW	PHL	Philippines	I -3
289	PALAGADAN	IDN	Indonesia	I -3
290	PALAGADAN	IDN	Indonesia	I -3
291	SARINGKIT	PHL	Philippines	I -3
292	TAPUL	PHL	Philippines	I -1
293	REKET BIDENG	IDN	Indonesia	I -3
294	NEAW-DUM	IDN	Indonesia	I -3

295	SUR-SANUR	IDN	Indonesia	I -1
296	BASTMATI	IDN	Indonesia	I -3
297	KHAO KUN MY LAY	THA	Tailand	I -3
298	LE	CHN	China	I -3
299	MYAYA NGA CHEIK	IDN	Indonesia	I -3
300	NGA CHEIK KYI	IDN	Indonesia	I -1
301	SHWE SABAR	MYS	Malaysia	I -3
302	TAPHAT CHEIK KAUKHNYIN	KHM	Cambodia	IV-1
303	TAUNG PAW NGA CHEIK	MMR	Myanmar	IV-2
304	ADONG(H)	PHL	Philippines	IV-2
305	HITAM PULUT(H)	IDN	Indonesia	I -3
306	NGA CHEIK PYU	IDN	Indonesia	I -3
307	E-GLAM	THA	Tailand	I -3
308	HAWM GAHB BANG	THA	Tailand	I -3
309	NIAW DAM MA GLEUA	THA	Tailand	I -3
310	NIAW DAM PLEUAK DAM	THA	Tailand	I -3
311	POTURU	MYS	Malaysia	I -3
312	PULUT TINDAL	IDN	Indonesia	I -1
313	PUTAN SUNDIG	BTN	Bhutan	I -1
314	TADONG PULUT	MYS	Malaysia	I -3
315	TADONG PULUT	MYS	Malaysia	I -3
316	RINDUYUH H	MYS	Malaysia	I -3
317	PULUT HITAM MELAKA	IDN	Indonesia	I -3
318	LEKATAN HITAM	IDN	Indonesia	I -3
319	NEP CAM	VNM	Vietnam	I -3
320	BIAW GUM GOO	THA	Tailand	I -3
321	NIAW DAM HAHNG	CHN	China	IV-2

322	NIAW DAM PLEUAK KHAO	THA	Tailand	Ⅰ-3
323	NIAW DAM PUANG	THA	Tailand	Ⅰ-3
324	NIAW PLEUAK DAM	THA	Tailand	Ⅰ-3
325	PAN NAM TOW	THA	Tailand	Ⅰ-3
326	PI MA LA GAW	THA	Tailand	Ⅰ-3
327	KAPUGUT	PHL	Philippines	Ⅰ-3
328	ADONG	PHL	Philippines	Ⅰ-3
329	TADONG	MYS	Malaysia	Ⅰ-3
330	KAM	BTN	Bhutan	Ⅰ-3
331	INABACA	PHL	Philippines	Ⅰ-3
332	NEANG YUORN	KHM	Cambodia	Ⅲ
333	MALAGKIT	PHL	Philippines	Ⅰ-3
334	SABIRARA MARAGKAT	PHL	Philippines	Ⅰ-3
335	KETAN HITAM 2	IDN	Indonesia	Ⅰ-3
336	KETAN IRENG 1	IDN	Indonesia	Ⅰ-3
337	MAUMERE 8	IDN	Indonesia	Ⅰ-3
338	PARE LOTONG 1	IDN	Indonesia	Ⅰ-3
339	PARE PULU LOTONG	IDN	Indonesia	Ⅰ-3
340	PARE PULU LOTONG 1	IDN	Indonesia	Ⅰ-3
341	ASE PUNU LEKLENG 1	IDN	Indonesia	Ⅰ-3
342	TADONG	MYS	Malaysia	Ⅰ-3
343	KAPUGUT	PHL	Philippines	Ⅲ
344	PADI PULUT HARANG	MYS	Malaysia	Ⅰ-3
345	ADONG	PHL	Philippines	Ⅰ-3
346	PULUT LAPAUNG	MYS	Malaysia	Ⅰ-3
347	TADONG	MYS	Malaysia	Ⅲ
348	MALIWARA	PHL	Philippines	Ⅰ-3

349	PULOT TA METEN	PHL	Philippines	Ⅰ-3
350	INABACA	PHL	Philippines	Ⅰ-3
351	MALAGKIT	PHL	Philippines	Ⅰ-3
352	우한 1	CHN	China	Ⅲ
353	우한 4	CHN	China	Ⅲ
354	백두산 1	CHN	China	Ⅳ-2
355	일본	JPN	Japan	Ⅲ
356	860036	JPN	Japan	Ⅲ
357	860062	JPN	Japan	Ⅲ
358	JUKU3	JPN	Japan	Ⅲ
359	JUKU4	JPN	Japan	Ⅲ
360	JUKU5	JPN	Japan	Ⅲ
361	JUKU6	JPN	Japan	Ⅲ
362	JUKU7	JPN	Japan	Ⅲ
363	JUKU8	JPN	Japan	Ⅲ
364	JUKU9	JPN	Japan	Ⅲ
365	Gagoshima1	JPN	Japan	Ⅲ
366	Gagoshima5	JPN	Japan	Ⅲ
367	Gagoshima8	JPN	Japan	Ⅲ
368	MALAGKIT 1	PHL	Philippines	Ⅰ-1
369	Gagoshima10	JPN	Japan	Ⅲ
370	Gagoshima13	JPN	Japan	Ⅲ
371	MALAGKIT 2	PHL	Philippines	Ⅰ-1
372	Gagoshima14	JPN	Japan	Ⅲ
373	Gagoshima16	JPN	Japan	Ⅲ
374	Gagoshima17	JPN	Japan	Ⅲ
375	Gagoshima18	JPN	Japan	Ⅲ

376	Gagoshima20	JPN	Japan	Ⅲ
377	Chokoto 14	JPN	Japan	Ⅰ-1
378	Ginkaragsare	KOR	Korea	Ⅳ-1
379	Hwalohung	KOR	Korea	Ⅰ-1
380	Jagwangdo	KOR	Korea	Ⅰ-1
381	PI 160766	CHN	China	Ⅰ-1
382	Tsital	KOR	Korea	Ⅳ-1
383	Ssalbyeo 1	KOR	Korea	Ⅲ
384	Ssalbyeo 2	KOR	Korea	Ⅰ-1
385	Ssalbyeo 3	KOR	Korea	Ⅲ
386	Ssalbyeo 4	KOR	Korea	Ⅳ-1
387	Ssalbyeo 5	KOR	Korea	Ⅲ
388	Ssalbyeo 6	KOR	Korea	Ⅰ-1
389	Ssalbyeo 7	KOR	Korea	Ⅲ
390	Ssalbyeo 8	KOR	Korea	Ⅳ-1
391	Ssalbyeo 9	KOR	Korea	Ⅳ-1
392	Ssalbyeo 10	KOR	Korea	Ⅲ
393	Ssalbyeo 11	KOR	Korea	Ⅲ
394	Ssalbyeo 12	KOR	Korea	Ⅲ
395	Ssalbyeo 14	KOR	Korea	Ⅱ
396	Ssalbyeo 15	KOR	Korea	Ⅲ
397	Ssalbyeo 16	KOR	Korea	Ⅰ-2
398	Ssalbyeo 17	KOR	Korea	Ⅳ-1
399	Ssalbyeo 18	KOR	Korea	Ⅳ-1
400	Ssalbyeo 19	KOR	Korea	Ⅳ-1
401	Ssalbyeo 20	KOR	Korea	Ⅰ-3
402	Ssalbyeo 21	KOR	Korea	Ⅰ-3

403	Ssalbyeo 22	KOR	Korea	Ⅲ
404	Ssalbyeo 23	KOR	Korea	Ⅳ-1
405	Ssalbyeo 24	KOR	Korea	Ⅳ-1
406	Ssalbyeo 25	KOR	Korea	Ⅰ-1
407	Ssalbyeo 26	KOR	Korea	Ⅰ-1
408	Ssalbyeo 27	KOR	Korea	Ⅲ
409	Ssalbyeo 28	KOR	Korea	Ⅲ
410	Ssalbyeo 29	KOR	Korea	Ⅳ-2
411	Ssalbyeo 31	KOR	Korea	Ⅰ-1
412	Ssalbyeo 33	KOR	Korea	Ⅳ-2
413	Ssalbyeo 34	KOR	Korea	Ⅰ-3
414	Ssalbyeo 35	KOR	Korea	Ⅳ-1
415	Ssalbyeo 36	KOR	Korea	Ⅰ-1
416	Ssalbyeo 37	KOR	Korea	Ⅳ-2
417	Ssalbyeo 38	KOR	Korea	Ⅳ-1
418	Ssalbyeo 39	KOR	Korea	Ⅳ-1
419	Ssalbyeo 40	KOR	Korea	Ⅳ-1
420	Ssalbyeo 41	KOR	Korea	Ⅳ-1
421	Ssalbyeo 41	KOR	Korea	Ⅳ-1
422	Ssalbyeo 43	KOR	Korea	Ⅰ-1
423	Ssalbyeo 45	KOR	Korea	Ⅳ-1
424	Ssalbyeo 46	KOR	Korea	Ⅳ-1
425	Ssalbyeo 47	KOR	Korea	Ⅳ-1
426	Ssalbyeo 48	KOR	Korea	Ⅳ-1
427	Ssalbyeo 49	KOR	Korea	Ⅳ-1
428	Ssalbyeo 51	KOR	Korea	Ⅲ
429	Ssalbyeo 52	KOR	Korea	Ⅳ-1

430	Ssalbyeo 53	KOR	Korea	Ⅳ-1
431	Ssalbyeo 54	KOR	Korea	Ⅰ-1
432	Ssalbyeo 55	KOR	Korea	Ⅰ-1
433	Ssalbyeo 56	KOR	Korea	Ⅳ-1
434	Ssalbyeo 57	KOR	Korea	Ⅳ-1
435	Ssalbyeo 58	KOR	Korea	Ⅳ-2
436	Ssalbyeo 59	KOR	Korea	Ⅳ-1
437	Ssalbyeo 61	KOR	Korea	Ⅰ-3
438	Ssalbyeo 62	KOR	Korea	Ⅰ-1
439	Ssalbyeo 63	KOR	Korea	Ⅰ-1
440	Ssalbyeo 64	KOR	Korea	Ⅰ-1
441	Ssalbyeo 65	KOR	Korea	Ⅲ
442	Murasaki-ine	JPN	Japan	Ⅳ-2
443	Murasaki-ine	JPN	Japan	Ⅲ
444	Tzushima-aka	JPN	Japan	Ⅲ
445	Nagasaki	JPN	Japan	Ⅲ
446	Nagasaki	JPN	Japan	Ⅰ-1
447	Nagasaki	JPN	Japan	Ⅰ-1
448	Miyazaki	JPN	Japan	Ⅰ-1
449	Miyazaki	JPN	Japan	Ⅰ-1
450	Miyazaki	JPN	Japan	Ⅳ-1
451	Miyazaki	JPN	Japan	Ⅳ-2
452	Miyazaki	JPN	Japan	Ⅳ-2
453	Miyazaki	JPN	Japan	Ⅱ
454	Miyazaki	JPN	Japan	Ⅰ-1
455	Miyazaki	JPN	Japan	Ⅰ-3
456	Miyazaki	JPN	Japan	Ⅰ-3

457	Miyazaki	JPN	Japan	Ⅲ
458	Miyazaki	JPN	Japan	Ⅲ
459	Gagoshima	JPN	Japan	Ⅲ
460	Gagoshima	JPN	Japan	Ⅰ-1
461	Gagoshima	JPN	Japan	Ⅲ
462	Gagoshima	JPN	Japan	Ⅱ
463	Ukinamisyusi	JPN	Japan	Ⅱ
464	Houmanjinzamai	JPN	Japan	Ⅰ-1
465	Houmanjinzamai	JPN	Japan	Ⅰ-1
466	Tobousi	JPN	Japan	Ⅰ-1
467	Douhousi	JPN	Japan	Ⅰ-1
468	Shiromuro	JPN	Japan	Ⅰ-1
469	Amamiya	JPN	Japan	Ⅰ-1
470	Shinoii	JPN	Japan	Ⅰ-1
471	Shinoii	JPN	Japan	Ⅰ-1
472	Nakatzu	JPN	Japan	Ⅰ-1
473	Karashita	JPN	Japan	Ⅰ-1
474	Karashita	JPN	Japan	Ⅰ-2
475	Karashita	JPN	Japan	Ⅰ-2
476	Chouyang	CHN	China	Ⅰ-1
477	Chouyang	CHN	China	Ⅳ-1
478	Reisui	JPN	Japan	Ⅰ-1
479	Akaho	JPN	Japan	Ⅰ-1
480	Xiaozhandabaimangshuidao	CHN	China	Ⅰ-1
481	Shengfangmi	CHN	China	Ⅰ-1
482	Nagohoaka	CHN	China	Ⅰ-1
483	Okinawazairai	JPN	Japan	Ⅱ

484	Biwakomochi	JPN	Japan	Ⅱ
485	Biwakomochi	JPN	Japan	Ⅱ
486	Hongxuenuo	CHN	China	Ⅰ-2
487	Akamai B	JPN	Japan	Ⅱ
488	Murasaki sankakuto	JPN	Japan	Ⅰ-2
489	Murasakiwase	JPN	Japan	Ⅱ
490	Murasaki ban	JPN	Japan	Ⅱ
491	Murasakiban chu	JPN	Japan	Ⅲ
492	Akaimo	JPN	Japan	Ⅰ-1
493	Tokushima akamai	JPN	Japan	Ⅰ-1
494	Clustered panicle	JPN	Japan	Ⅰ-1
495	Tsushima aka	JPN	Japan	Ⅰ-1
496	Zhongshu sanbailigengdao	CHN	China	Ⅰ-1
497	Seenatty	LKA	Sri Lanka	Ⅰ-1
498	Seenatty	LKA	Sri Lanka	Ⅰ-1
499	Zaoshu lanhou xiandao	CHN	China	Ⅰ-1
500	Zaoshu pingyangbai xiandao	CHN	China	Ⅰ-1
501	Zhongshu hongzuiyu gengdao	CHN	China	Ⅰ-1
502	Zhongshu hongke gengdao	CHN	China	Ⅰ-1
503	Zhongshu mangke gengdao	JPN	Japan	Ⅲ
504	Wanshu dahongzao gengdao	CHN	China	Ⅰ-1
505	Ronghedao	CHN	China	Ⅰ-1
506	Ronghedao1	CHN	China	Ⅰ-1
507	Carolina	PAK	Pakistan	Ⅰ-1
508	Chouyang1	CHN	China	Ⅳ-2
509	East African white	JPN	Japan	Ⅱ
510	Shirori	JPN	Japan	Ⅲ

511	Chouyang2	CHN	China	Ⅳ-1
512	Hatadani	JPN	Japan	Ⅲ
513	Basmati	IDN	Indonesia	Ⅰ-1
514	Bawiash murali	JPN	Japan	Ⅲ
515	Black seenatty	LKA	Sri Lanka	Ⅰ-3
516	Black seenatty	LKA	Sri Lanka	Ⅰ-1
517	Ronghedao2	CHN	China	Ⅰ-1
518	Zhongshu hongzuiyu gengdao	CHN	China	Ⅰ-1
519	Ssalbyeo 60	KOR	Korea	Ⅳ-1
520	Akamai 65	JPN	Japan	Ⅰ-1
521	Akamai 66	JPN	Japan	Ⅰ-1
522	Akamai 67	JPN	Japan	Ⅰ-3
523	Shirori	JPN	Japan	Ⅲ
524	Shirori	JPN	Japan	Ⅲ
525	Ssalbyeo 61	KOR	Korea	Ⅳ-1
526	Akamai 68	JPN	Japan	Ⅰ-1
527	Ssalbyeo 62	KOR	Korea	Ⅳ-1
528	Shirori	JPN	Japan	Ⅲ
529	Akamai 69	JPN	Japan	Ⅰ-1
530	Akamai 70	JPN	Japan	Ⅰ-1
531	Akamai 71	JPN	Japan	Ⅰ-1
532	Akamai 72	JPN	Japan	Ⅲ
533	R5-Mixed A	JPN	Japan	Ⅲ
534	R5-Mixed B	CHN	China	Ⅳ-2
535	R5-Mixed C	CHN	Japan	Ⅰ-2
536	R82-Mixed	JPN	Japan	Ⅰ-1
537	R98-Mixed	JPN	Japan	Ⅰ-2

538	R120-Mixed, Awned	JPN	Japan	Ⅰ-1
539	R123-Mixed, Colored	LKA	Sri Lanka	Ⅰ-1
540	R139-Mixed	JPN	Japan	Ⅰ-1
541	R148-Mixed A	JPN	Japan	Ⅱ
542	R148-Mixed B	JPN	Japan	Ⅰ-1
543	R89-Mixed B	JPN	Japan	Ⅰ-1
544	R150-Mixed	JPN	Japan	Ⅰ-1
545	R29-Mixed	KOR	Korea	Ⅲ
546	R34-Mixed	KOR	Korea	Ⅳ-1
547	R101-Mixed	CHN	China	Ⅰ-1
548	R164-Mixed	JPN	Japan	Ⅰ-1
